# Use of the Vascular Overload Index to Predict Cardiovascular Disease in a Rural Population of China

**DOI:** 10.1155/2022/5289122

**Published:** 2022-12-14

**Authors:** Chang Wang, Chuning Shi, Songyue Liu, Danxi Geng, Yingxian Sun

**Affiliations:** Department of Cardiovascular Medicine, The First Hospital of China Medical University, Shenyang, Liaoning 110001, China

## Abstract

**Objective:**

To explore the relationship between vascular overload index (VOI) and cardiovascular disease (CVD) in rural population and find effective ways to prevent cardiovascular disease in rural low-income populations.

**Methods:**

The data for this study was obtained from a large cohort study called the Northeast China Rural Cardiovascular Health Study (NCRCHS) conducted in 2013 and followed up during 2015-2018. 10,174 subjects completed at least one follow-up visit. Cox regression equation was used to explore whether VOI and cardiovascular disease were independently related. The Kaplan-Meier curves were used to calculate the cumulative incidence of any adverse outcome, and the log-rank test and restrict mean survival analysis were used to compare group differences. Reclassification and discrimination statistics were used to determine whether VOI could strengthen the ability of the model to predict CVD events.

**Results:**

The prevalence of CVD in the VOI quartiles was 1.92%, 3.96%, 5.42%, and 11.34% for Q1–Q4, respectively (*P* for trend <0.001). After adjusting for multiple confounders, there was a 2.466-fold increased risk of CVD when comparing the highest and lowest groups. Besides, this study found that for every standard deviation increase, the results still exist. The risk of cardiovascular disease increased by 1.358-fold in this model. The restrict mean survival analysis results show that with the increase of VOI, the restrict mean survival time (RMST) within 5 years gradually became shorter. Reclassification and discrimination statistics indicated that VOI significantly enhanced the ability to estimate CVD events within 4 years.

**Conclusion:**

Analyses showed that VOI was significantly associated with CVD. VOI is a simple and accurate prognostic marker of CVD risk, which has the potential ability to improve the risk stratification of CVD.

## 1. Introduction

Cardiovascular disease (CVD) is a condition with one of the highest morbidity and mortality rates worldwide, contributing to the global disease burden and posing a great challenge to public health [1, 2]. In China, the number of CVD-related deaths increased from 2.51 million in 1990 to 3.97 million in 2016, when the prevalent cases reached nearly 94 million [3]. The incidence of CVD is particularly high in low-income rural areas of China [4]. It is critical to screen this and other high-risk groups in order to take appropriate preventive measures against this disease.

Arterial stiffness, which can cause the formation of atherosclerotic plaques and eventually lead to the narrowing of blood vessels, is a major risk factor for CVD [5]. Early detection of vascular elasticity has important clinical significance for the prevention and treatment of cardiovascular events. Franklin and Weber proposed the concept of vascular overload, defined as increased peripheral arterial resistance, arteriosclerosis, and the early occurrence of pulse reflection waves which work together to alter cardiovascular function and structure [6]. The vascular overload index (VOI) is a measure of vascular elasticity that considers the progressive elasticity of the vessel wall, arterial stiffness, and arterial resistance as well as systolic blood pressure. This index is used to standardize the blood vessel load during circulation and can evaluate an individual's risk of vascular disease.

VOI was associated with new-onset stroke in an elderly hypertensive population [7]. The Framingham study confirmed that systolic blood pressure is independently associated with stroke risk [8]. The VOI calculation method combines the common characteristics of systolic and diastolic blood pressure and reflects the vascular load. However, the ability of VOI to prognose CVD in the general population remains largely unexplored. The current study is based on data from 10,174 participants in the Northeast China Rural Cardiovascular Health Study (NCRCHS) who had a median follow-up time of 4.65 years. The aim was to examine whether VOI was independently associated with CVD in a rural population in China.

## 2. Methods

### 2.1. Study Population

The data for this study was obtained from a large cohort study called the Northeast China Rural Cardiovascular Health Study (NCRCHS) which was described in detail previously [9, 10]. After excluding pregnant women and patients with mental disorders or cancer, a total of 11,956 patients were recruited for the NCRCHS in 2013. Between 2015 and 2018, all subjects were invited to participate in follow-up, and 10,700 eventually agreed to follow-up studies. A total of 10,349 (96.7%) patients completed at least one follow-up visit. In the current study, we excluded patients with missing anthropometric data (*n* = 93). Besides, we excluded patients with missing blood biochemical parameters (*n* = 82). Finally, 10,174 patients were included in the data analysis.

### 2.2. Data Collection

The cardiologists and nurses responsible for administering the survey received specialized training prior to the study. The survey included information on demographics, health-related behaviors, anthropometric parameters, CVD history, dietary intake, family history of diabetes, educational attainment, annual household income, and drug use over the past two weeks. After the subjects rested in a sitting position for at least 5 minutes, blood pressure was measured by two trained staff members using an automated electronic sphygmomanometer (HEM-907; Omron, Tokyo, Japan) with the arm supported at the level of the heart, and three measurements were recorded for each subject. Average readings of repeated measures were used for sample analysis. The subjects wore light clothing for anthropometric measurements. A calibrated electronic weight scale (accurate to 0.1 kg), portable distance meter (accurate to 0.1 cm), and nonelastic tape measure (accurate to 0.1 cm) were used to take two measurements of each subject's weight, height, and waist circumference. The average of these measurements was used for analysis. Fasting (12 hours overnight) blood samples were obtained from each subject by venipuncture and collected in EDTA tubes. Within 1 hour, the plasma was separated, frozen at -20°C, and sent to a qualified laboratory for testing. Biochemical analysis was performed using an Olympus AU 640 automatic analyzer (Olympus, Kobe, Japan) to obtain fasting blood glucose, total cholesterol (TC), low-density lipoprotein cholesterol (LDL-C), high-density lipoprotein cholesterol (HDLC), triglyceride (TG), uric acid, serum creatinine, and other routine blood biochemical parameters. All laboratory equipment was calibrated, and the samples were analyzed in blinded replicates.

### 2.3. Definitions

VOI was calculated using the systolic and diastolic blood pressure as follows: VOI (mmHg) = 1.33 × SBP − 0.33 × DBP − 133.3 [6]. Hypertension was defined as a systolic blood pressure > 140 mmHg, a diastolic blood pressure > 90 mmHg, or having taken antihypertensive drugs for the prior 2 weeks [11]. BMI was defined as weight divided by height squared. Diabetes was defined as a rapid blood glucose test > 7.0, a history of any diabetes diagnosis, or the receipt of any hypoglycemic therapy in the prior 2 weeks [12]. Exercise is defined as moderate intensity exercise (equivalent to walking) ≥30 minutes and ≥3 times a week. Current smoking is defined as at least one cigarette a day [13]. Current drinking is defined as any amount of alcohol at least once a week [4].

### 2.4. Determination of the Primary Endpoint

All available clinical information about a possible diagnosis or death was collected from medical records and death certificates. The material was independently reviewed and adjudicated by the Incident Committee. Stroke is defined by the World Health Organization (WHO) as a rapidly developing focal (or global) brain dysfunction that lasts >24 hours (unless interrupted by surgery or death) and has no apparent nonvascular cause diagnosed by a neurologist after examining computed tomography and magnetic resonance imaging data [14]. CHD included a diagnosis of angina pectoris, myocardial infarction (MI), revascularization surgery, and CHD-related mortality requiring hospitalization [15]. CVD was defined as stroke or coronary heart disease (CHD).

### 2.5. Data Analysis

Continuous variables are expressed as means ± standard deviation, and categorical variables are expressed as frequencies (percentages). Baseline data were assessed for differences between groups using the Student's *t*-test and ANOVA for continuous variables or the chi-square test for categorical variables. Before the *t*-test, the homogeneity of variance test shall be carried out, and the *t*-test or the corrected *t*-test shall be selected according to the test results. After adjusting for age, sex, BMI, exercise, current smoking, current drinking, eGFR, TC, TG, HDL-C, DM, HTN, and CVD history, VOI was defined as a continuous variable (per SD) and divided into four quartiles. The number of digits was included in the Cox regression equation to explore whether VOI and CVD were independently related. The results are presented with HR and 95% confidence intervals. The threshold for statistical significance was defined as *P* < 0.05. K-M curves were used to calculate the cumulative incidence of any adverse outcomes, and log-rank tests were used to compare the differences. In order to reflect the differences between groups, this study calculated the restricted mean survival time within five years and visualized the results. The reclassification improvement indices, NRI and IDI, were used to compare the ability of a traditional risk factor-only model and a VOI+traditional risk factor model to predict CVD events. In this study, 2-tailed*P*value < 0.05 is statistically significant for the analysis. All data analyses were performed using SPSS 25.0 and R statistical software (http://www.r-project.org, R Foundation).

## 3. Results

**Figure 1:**
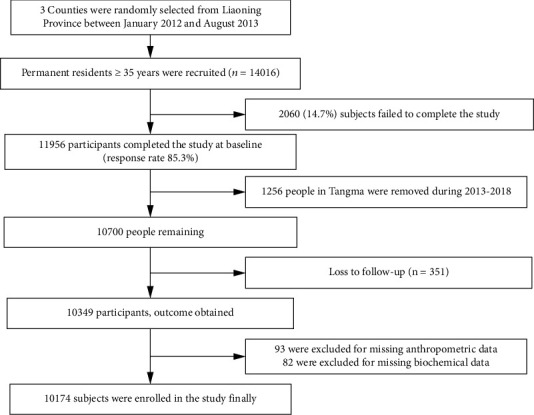
Flowchart describing the recruitment process and generation of study population.

The 10,174 subjects included in the study had a mean age of 53.76 ± 10.48 years, and 46.4% were male. The flowchart describing the recruitment process and generation of study population is shown in Figure 1. The characteristics of the subjects in each VOI quartile were compared at baseline. Individuals with a higher VOI were older and had significantly higher BMI and TC, TG, FPG, and HDL-C levels (*P* < 0.05). Subjects with a higher VOI were also more likely to lead an unhealthier lifestyle, as shown by their increased rates of smoking and drinking alcohol (Table 1).

**Figure 2:**
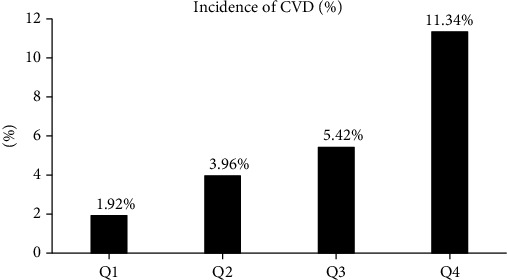
The prevalence of CVD by VOI quartile.

The prevalence of CVD in the VOI quartiles was 1.92%, 3.96%, 5.42%, and 11.34% for Q1–Q4, respectively (*P* for trend <0.001) (Figure 2). To explore the relationship between VOI and adverse outcomes of CVD, Cox regression analysis was conducted (Table 2). In model 1, which had no adjustment for confounders, subjects in the highest quartile had a 6.218-fold higher risk of CVD than those in the lowest quartile. In model 2, which is controlled for age, sex, BMI, exercise, current smoking, current drinking, eGFR, TC, TG, HDL-C, DM, HTN, and CVD history-related confounders, subjects in the highest VOI quartile still had a higher risk of CVD than those in the lowest VOI quartile. The VOI HR was 2.466 (1.581–3.846, *P* < 0.001). When VOI was assessed as a continuous variable, the risk of CVD increased by 1.358 for each increase in SD.

**Figure 3:**
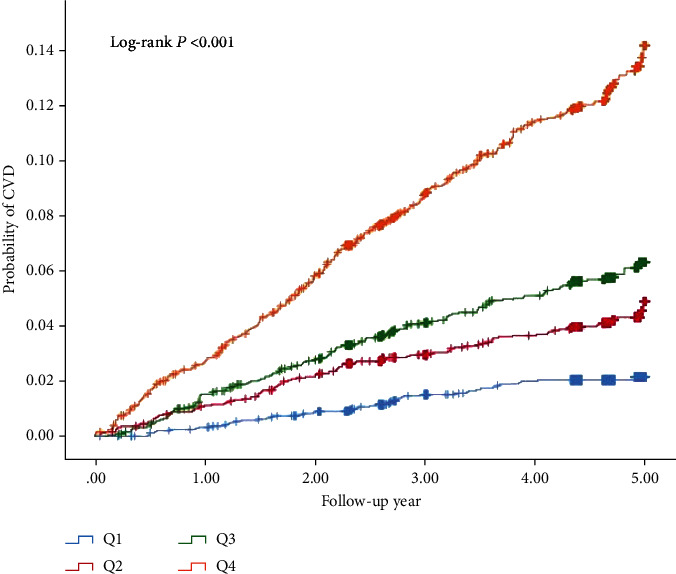
Unadjusted Kaplan-Meier curves for incident adverse events by VOI quartile.

**Figure 4:**
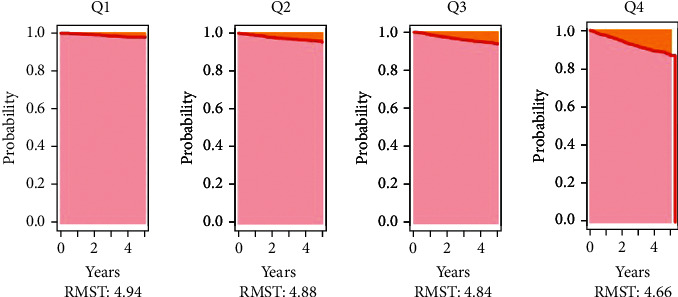
Restricted mean survival (RMS) curves for quartiles of VOI in cohorts.

The Kaplan-Meier curves of the four groups are shown in Figure 3. The cumulative incidence risk of CVD was significantly greater in the Q4 group than in the Q1 group (*P* for log-rank <0.001). The restrict mean survival analysis results show that with the increased of VOI, the restrict mean survival time (RMST) within 5 years gradually became shorter (Table S1). The restrict mean survival time from Q1 to Q4 is represented by RMST1: 4.945 years (95% CI: 4.929-4.961), RMST2: 4.800 years (95% CI: 4.995-4.905), RMST3: 4.840 years (95% CI: 4.811-4.868), and RMST4: 4.633 years (4.623-4.703), respectively. Compared with the reference (Q1), there are group differences between reference and Q2, Q3, Q4 (*P* < 0.001) (Table S2). RMS curves showed the highest slope in Q4, indicating the shortest RMST in Q4 participants (Figure 4). Reclassification and discrimination statistics for CVD were conducted within 4 years and stratified by VOI (Table 3). VOI was also added to traditional models to determine whether prediction performance improved. IDI and NRI values showed that adding VOI to the model predicted a significantly higher incidence of CVD within 4 years. Finally, the stratified analysis verified that the results are robust (Table 4).

## 4. Discussion

This study showed that VOI is an independent risk factor for CVD events in a representative rural population of China, regardless of whether VOI is defined as a categorical or continuous variable. The findings remained significant when many traditional risk factors were included in the regression equation. The highest-grade VOI was associated with a 2.466-fold higher risk of developing CVD than the lowest-grade VOI. The results also showed that VOI significantly improved CVD risk stratification in the general population. Stratified analysis showed that this finding remained stable among different subpopulations.

Franklin and Weber proposed the concept of vascular overload and defined pathological changes and clinical events as effective blood pressure increments, with VOI serving as an objective index for evaluation [6]. VOI has been associated with ischemic stroke in elderly hypertensive patients [7]. Higher VOI in hypertensive patients is also associated with increased carotid artery media thickness. For every 10 mmHg increase in VOI, there is a 0.24% decrease in vascular endothelial diastolic function, a 1.95 g/m^2^ increase in LVMI, and a 0.036 mmHg increase in carotid artery media thickness [16]. A large metastudy showed that VOI is more often associated with hypertensive cardiovascular events than SBP [17]. However, prior to the current study, the association between VOI and CVD-related adverse outcomes was not yet assessed in the general population. The current study confirmed that VOI was independently associated with CVD in a representative rural population of China. This finding suggests that high-risk groups should be screened for CVD in economically underdeveloped rural areas in order to effectively reduce the disease burden.

After adding new markers to an existing model, it is necessary to calculate NRI and IDI to determine whether the new model has improved predictive power [18]. The current study showed that when VOI was added to traditional risk models, the model had an improved ability to predict CVD. NRI and IDI values also suggested that adding VOI significantly improved risk stratification for CVD.

Vascular damage, which includes endothelial dysfunction, lipid deposition, increased arterial stiffness, and the formation of atherosclerotic plaques, is the primary cause of CVD [19]. High low-density lipoprotein cholesterol (LDL-C) and serum uric acid (SUA) levels are risk factors for endothelial dysfunction and vascular ageing [20]. The reduced elasticity of large arteries is an early manifestation of arterial wall sclerosis [21]. Thus, early detection of vascular elasticity is critical for the effective prevention and treatment of cardiovascular events. The Framingham study has paid more attention to the impact of systolic blood pressure on adverse outcomes and less attention to the effect of diastolic blood pressure [8]. Vascular elasticity, arterial stiffness, and small vessel resistance are all determined by diastolic blood pressure. While VOI is primarily based on systolic blood pressure, diastolic blood pressure is also taken into account. This suggests that vascular overload, rather than blood pressure, may be a better indicator of adverse cardiovascular outcomes.

The current study provides a reference for the use of VOI as a clinical marker for individuals at high risk of CVD and suggests that this metric should be applied to the general population of rural China. Given that the required data and calculation methods are easy to obtain, VOI is a practical tool to aid in identifying CVD risk.

This study also has some limitations. Firstly, since VOI is a parameter that reflects arterial stiffness, pulse wave velocity and the augmentation index were not used as validation methods, which may reduce the persuasiveness of the results. Secondly, drugs have the effect of protecting vascular endothelium which will change the specific value of VOI. The subjects recruited in this study have a history of taking drugs, and the influence of some drugs cannot be excluded. Thirdly, some blood biochemical indicators are biomarkers of atherosclerosis, such as high-sensitivity C-reactive protein, which are related to atherosclerosis and cardiovascular risk. This is not measured in our current study. Fourthly, the study population was recruited from rural China and may be different from populations in developed or high-income areas and needs to be verified by different population cohorts. However, these limitations do not affect the implications of this study for future strategies to prevent CVD.

## 5. Conclusion

The results of the current study suggest that VOI is a simple and accurate prognostic marker of CVD risk. These findings provide new prospective data showing that VOI is positively associated with CVD incidence among adults in rural China. The results further show that VOI has the potential ability to improve the risk stratification of CVD.

## Figures and Tables

**Table 1:** Baseline characteristics of participants' overall and by VOI quartile.

Variables	Overall	VOI by quartiles				*P* value
		Q1 (*n* = 2,502)	Q2 (*n* = 2,551)	Q3 (*n* = 2,545)	Q4 (*n* = 2,576)	
Age (y)	53.76 ± 10.48	49.04 ± 9.09	51.34 ± 9.70	54.84 ± 10.10	59.68 ± 9.80	<0.001
Sex (male, %)	4,723 (46.4)	949 (37.9)	1,228 (48.1)	1,276 (50.1)	1,270 (49.3)	<0.001
Ethnicity (Han, %)	9,577 (94.1)	2,369 (94.8)	2,407 (94.5)	2,385 (93.7)	2,416 (94.0)	0.360
SBP	142.12 ± 23.44	116.22 ± 6.80	131.41 ± 4.05	145.87 ± 5.45	174.17 ± 16.52	<0.001
DBP	82.14 ± 11.75	72.50 ± 7.23	79.46 ± 7.64	84.54 ± 9.43	91.78 ± 12.35	<0.001
BMI	24.83 ± 3.68	23.56 ± 3.31	24.74 ± 3.81	25.25 ± 3.66	25.74 ± 3.56	<0.001
Exercise (*N*, %)	2,141 (21.0)	455 (18.2)	457 (17.9)	593 (23.3)	636 (24.7)	<0.001
Education status						<0.001
Primary	5,146 (50.5)	1,056 (42.4)	1,186 (46.7)	1,282 (50.6)	1,578 (61.6)	
Middle	4,091 (40.2)	1,189 (47.7)	1,096 (43.1)	1,012 (39.9)	794 (31.0)	
High	937 (9.2)	246 (9.9)	259 (10.2)	242 (9.5)	190 (7.4)	
Current smoking (*N*, %)	3,606 (35.4)	838 (33.5)	949 (37.2)	940 (36.9)	879 (34.1)	0.007
Current drinking (*N*, %)	2,324 (22.8)	411 (16.4)	628 (24.6)	620 (24.4)	665 (25.8)	<0.001
eGFR (ml/min)	93.56 ± 15.44	95.72 ± 13.38	95.48 ± 15.66	92.98 ± 15.60	90.13 ± 16.24	<0.001
TG (mmol)	1.60 ± 1.45	1.31 ± 1.03	1.55 ± 1.41	1.70 ± 1.59	1.82 ± 1.64	<0.001
TC (mmol)	5.24 ± 1.09	4.94 ± 0.98	5.21 ± 1.06	5.32 ± 1.09	5.50 ± 1.14	<0.001
HDL-C (mmol)	1.42 ± 0.39	1.37 ± 0.35	1.42 ± 0.38	1.43 ± 0.39	1.46 ± 0.43	<0.001
FPG (mmol)	5.88 ± 1.61	5.54 ± 1.22	5.74 ± 1.31	6.03 ± 1.74	6.23 ± 1.94	<0.001
HTN (*N*, %)	5,176 (50.9)	93 (3.7)	360 (14.1)	2,147 (84.4)	2,576 (100)	<0.001
Diabetes (*N*, %)	433 (4.3)	41 (1.6)	71 (2.8)	137 (5.4)	184 (7.1)	<0.001
CVD history (*N*, %)	805 (7.9)	114 (4.6)	138 (5.4)	208 (8.2)	345 (13.4)	<0.001

Abbreviations: SBP: systolic blood pressure; DBP: diastolic blood pressure; BMI: body mass index; eGFR: estimated glomerular filtration rate; TG: triglycerides; TC: total cholesterol; HDL: high-density lipoprotein cholesterol; FPG: fasting blood glucose; CVD history: history of cardiovascular disease.

**Table 2:** Multivariate Cox regression analyses for VOI and CVD.

Variables	Events	HR (95% CI) crude model	*P* value	HR (95% CI) model 1	*P* value	HR (95% CI) model 2	*P* value
VOI (per SD change)	579	1.756 (1.645–1.876)	<0.001	1.446 (1.344–1.557)	<0.001	1.358 (1.234–1.494)	<0.001
Quartiles of VOI							
Q1	48	1		1		1	
Q2	101	2.079 (1.474–2.931)	<0.001	1.773 (1.256–2.502)	0.001	1.671 (1.177–2.371)	0.004
Q3	138	2.872 (2.068–3.989)	<0.001	1.996 (1.431–2.783)	<0.001	1.566 (1.019–2.406)	0.041
Q4	292	6.218 (4.582–8.439)	<0.001	3.312 (2.413–4.547)	<0.001	2.466 (1.581–3.846)	<0.001
*P* for trend			<0.001		<0.001		<0.001

Abbreviations: VOI: vascular overload index; HR: hazard ratio; 95% CI: 95% confidence interval.

**Table 3:** Reclassification and discrimination statistics for adverse outcomes experienced within 4 years by VOI.

Model	NRI (95% CI)	IDI	
		Estimate	*P*
Conventional risk factors	Reference	Reference	
Conventional risk factors+VOI	0.0580 (0.0081–0.1087)	0.0055	<0.001

Abbreviations: VOI: vascular overload index; NRI: net reclassification improvement; NRI: integrated discrimination improvement; 95% CI: 95% confidence interval.

**Table 4:** Subgroup analyses for the impact of VOI on the risk of adverse outcomes.

	*N*	HR	95% CI	*P* value
Age (years)				
<55	5619	1.024	(1.017–1.031)	<0.001
≥55	4555	1.01	(1.007–1.014)	<0.001
Sex				
Male	4723	1.014	(1.009–1.019)	<0.001
Female	5451	1.007	(1.002–1.012)	0.004
BMI (kg/m^2^)				
<28	8351	1.01	(1.006–1.014)	<0.001
≥28	1823	1.013	(1.006–1.019)	<0.001
DM				
Yes	432	1.002	(0.990–1.013)	0.078
No	9741	1.012	(1.008–1.015)	<0.001

Abbreviations: BMI: body mass index; DM: diabetes; HR: hazard ratio; 95% CI: 95% confidence interval.

## Data Availability

The datasets used and/or analyzed during the current study do not contain identifiable data and are available from the corresponding author on reasonable request.

## Abbreviations

CVD: Cardiovascular disease
VOI: Vascular overload index
BMI: Body mass index
eGFR: Estimated glomerular filtration rate
TC: Total cholesterol
TG: Triglyceride
HDL-C: HDL cholesterol
DM: Diabetes mellitus
HTN: Hypertension.

## Supplementary Materials

Supplementary Materials Table S1: the restrict mean survival analysis results show that with the increased of VOI, the restrict mean survival time (RMST) within 5 years gradually became shorter. The restrict mean survival time from Q1 to Q4 is represented by RMST1: 4.945 years (95% CI: 4.929-4.961), RMST2: 4.800 years (95% CI: 4.995-4.905), RMST3: 4.840 years (95% CI: 4.811-4.868), and RMST4: 4.633 years (4.623-4.703), respectively. Supplementary description of Table S2: compared with the reference (Q1), there are group differences between reference and Q2, Q3, and Q4 (*P* < 0.001).
