# Supplementary material for: Use of the Vascular Overload Index to Predict Cardiovascular Disease in a Rural Population of China
Source: Biomed Res Int. 2022 Dec 14;2022:5289122. doi: 10.1155/2022/5289122 (PMC9771649; doi:10.1155/2022/5289122)
Supplement: Supplementary Materials — Table S1: the restrict mean survival analysis results show that with the increased of VOI, the restrict mean survival time (RMST) within 5 years gradually became shorter. The restrict mean survival time from Q1 to Q4 is represented by RMST1: 4.945 years (95% CI: 4.929-4.961), RMST2: 4.800 years (95% CI: 4.995-4.905), RMST3: 4.840 years (95% CI: 4.811-4.868), and RMST4: 4.633 years (4.623-4.703), respectively. Supplementary description of Table S2: compared with the reference (Q1), there are group differences between reference and Q2, Q3, and Q4 (P < 0.001). [file 5289122.f1.docx]

Table S1. The restricted mean survival time within five years distinguished by quartiles of VOI

| Quartiles of VOI | RMST (years) | 95%CI |
| --- | --- | --- |
| Q1 | 4.945 | (4.929-4.961) |
| Q2 | 4.880 | (4.885-4.905) |
| Q3 | 4.840 | (4.811-4.868) |
| Q4 | 4.663 | (4.623-4.703) |

Abbreviations: VOI - vascular overload index, RMST - Restricted Mean Survival Time; 95%CI – 95% confidence interval

Table S2. Between group contrast in restricted mean survival time distinguished by quartiles of VOI

| Variables | Value | 95%CI | *p* |
| --- | --- | --- | --- |
| RMST1-RMST2 | 0.065 | (0.035-0.095) | *<0.001* |
| RMST1/RMST2 | 1.013 | (1.007-1.020) | *<0.001* |
| RMTL1/RMTL2 | 0.458 | (0.319-0.659) | *<0.001* |
| RMST1-RMST3 | 0.105 | (0.073-0.138) | *<0.001* |
| RMST1/RMST3 | 1.022 | (1.015-1.029) | *<0.001* |
| RMTL1/RMTL3 | 0.343 | (0.242-0.485) | *<0.001* |
| RMST1-RMST4 | 0.282 | (0.239-0.325) | *<0.001* |
| RMST1/RMST4 | 1.06 | (1.051-1.070) | *<0.001* |
| RMTL1/RMST4 | 0.163 | (0.118-0.225) | *<0.001* |

Abbreviations: RMST1- Q1 subjects’ restricted mean survival time; RMST2- Q2 subjects’ restricted mean survival time; RMST3- Q3 subjects’ restricted mean survival time; RMST4- Q4 subjects’ restricted mean survival time; RMTL1- Q1 subjects’ restricted mean time lost; RMTL2- Q2 subjects’ restricted mean time lost; RMTL3- Q3 subjects’ restricted mean time lost; RMTL4- Q4 subjects’ restricted mean time lost; 95%CI – 95% confidence interval
